# Case of Alarming Syncope from the Admission of Air into a Vein during an Amputation at the Shoulder-joint

**Published:** 1844-04-06

**Authors:** Bransby B. Cooper

**Affiliations:** Surgeon to Guy’s Hospital


*Case of alarming Syncope from the Admission of Air into a Vein during an Amputation at the Shoulder-joint.* By Bransby B. Cooper, Esq. F.R.S. Surgeon to Guy's Hospital.

The patient, a female, nineteen years of age, was admitted on the 17th of May, 1843, under the care of the author, for enlargement of the middle third of the humerus. She had suffered pain in the seat of the disease for eight months, but the swelling did not commence till six weeks before her admission into the hospital. From the history and the examination of the arm, the tumor was considered to be a malignant disease of the bone, and amputation at the shoulder-joint was resolved upon. This operation was performed on the 23d of May. The arm was removed in less than a minute, and with very little loss of blood. When the limb had been severed from the body, the patient, who had borne the operation with great fortitude, expressed her thankfulness in a firm and audible voice The subclavian artery was immediately secured, but its compression still retained upon the first rib, as there were small vessels requiring ligature. The author then proceeded to remove a gland, which was somewhat enlarged, from the axilla; and while dissecting it from its cellular attachments, he distinctly heard a peculiar gurgling noise, like air escaping with fluid from a narrow-necked bottle; and at the same instant the patient fell into a state of collapse, threatening immediate dissolution: the countenance was deadly pale; pupils fixed, and unobedient to light; the pulse quick, small, and fluttering, although at intervals regular; the respiration was irregular, being hurried and feeble, and attended occasionally with a deep sigh. The patient was directly placed in the horizontal posture, the flap brought over the wound, and retained by plaister; and various stimulants were administered. An hour elapsed before she was sufficiently recovered to be removed from the operating theatre. Upon being placed in bed, she passed her fæces and urine involuntarily. When the reaction was coming on, she uttered a continued whining cry, and maintained a constant motion of alternate flexion and extension of the right leg, while the left remained perfectly quiescent, and seemed insensible to feeling, as well as motionless. She complained also of pain running up the right side of the head and neck. For several days she remained with her eyes closed. The lower extremities in the same condition as described, and the pulse very frequent. It was found necessary to give her opiates at different times, which relieved her general restlessness, and procured sleep. On the fourth day, the left leg was also affected with involuntary contractions; but these subsided on the following day. On the 25th day she was able to leave her bed. The motions of the right leg had ceased at this time, but she complained of great numbness and loss of power in the left leg. On the 3d of July she was sufficiently recovered to leave the hospital, having no other unfavourable symptom but a slight dragging of the left leg. The author concluded his paper by adding remarks on the consequences of air being admitted into the veins, and pointed out the resemblance between the symptoms in his case and those presented in other similar cases upon record, as well as in experiments upon the lower animals, made to elucidate the subject. He drew attention to the different effect produced in man compared with brutes by the admission of air, owing to the influence of mental agitation on the motions of the heart in the former. He also dwelt on the various modes of death in such cases, according as the air introduced distended and paralysed the walls of the right cavities of the heart, or was sent onwards, mixed with the blood, to the lungs, or was transmitted by the arteries to the brain; and he ended by offering some practical remarks on the best mode of guarding patients from such dangerous occurrences in operations about the neck and shoulder.—*Lond. Med. Gaz.*, Dec. 22, 1843.

